# A new default restraint library for the protein backbone in *Phenix*: a conformation-dependent geometry goes mainstream

**DOI:** 10.1107/S2059798315022408

**Published:** 2016-01-01

**Authors:** Nigel W. Moriarty, Dale E. Tronrud, Paul D. Adams, P. Andrew Karplus

**Affiliations:** a Physical Biosciences, Lawrence Berkeley National Laboratory, Berkeley, CA 94720, USA; b Department of Biochemistry and Biophysics, Oregon State University, Corvallis, OR 97377, USA; c Department of Bioengineering, University of California Berkeley, Berkeley, CA 94720, USA

**Keywords:** covalent geometry restraints, crystallographic refinement, protein structure, validation, *Phenix*

## Abstract

Chemical restraints are a fundamental part of crystallographic protein structure refinement. In response to mounting evidence that conventional restraints have shortcomings, it has previously been documented that using backbone restraints that depend on the protein backbone conformation helps to address these shortcomings and improves the performance of refinements [Moriarty *et al.* (2014 ▸), *FEBS J.* **281**, 4061–4071]. It is important that these improvements be made available to all in the protein crystallography community. Toward this end, a change in the default geometry library used by *Phenix* is described here. Tests are presented showing that this change will not generate increased numbers of outliers during validation, or deposition in the Protein Data Bank, during the transition period in which some validation tools still use the conventional restraint libraries.

## Abstract 2

The default geometry restraints used in *Phenix* for the protein backbone have been upgraded to account for the known conformation-dependencies of bond angles and lengths.

Since the mid-1980s (see, for example, Scarsdale *et al.*, 1983 ▸; Schäfer *et al.*, 1984 ▸), quantum-mechanics calculations have shown that the backbone bond angles of dipeptide model compounds vary substantially with protein conformation (*i.e.* the φ and ψ torsion angles). This was later confirmed to occur in proteins (Jiang *et al.*, 1995 ▸; Karplus, 1996 ▸), but remained a little known reality until it was suggested that accounting for this behaviour might resolve a controversy about how best to handle restraints in protein crystallographic refinements (Karplus *et al.*, 2008 ▸). Shortly thereafter, an analysis was carried out that codified how backbone bond lengths and angles were observed to change with conformation in a large set of protein crystal structures solved at 1 Å resolution or better (Berkholz *et al.*, 2009 ▸). It was concluded that the bond-angle variations were reliably determined and substantial in size, but that the bond-length variations were less reliably determined and also so small as to be of little importance in modeling accuracy. In an accompanying highlight article (Dauter & Wlodawer, 2009 ▸) it was stated ‘Hopefully, the structural biology community will soon adopt the ideas’.

From the above results, a formalized empirical conformation-dependent library (CDL) for *trans*-peptide backbone restraints was developed (Tronrud *et al.*, 2010 ▸) and was tested in protein crystallographic refinement using the *TNT* (Tronrud *et al.*, 1987 ▸) and *SHELXL* (Sheldrick, 2008 ▸) refinement programs. For the test-case structures studied, these tests showed that using the CDL (v.1.2) instead of a common conventional restraint library (Engh & Huber, 2001 ▸) led to much lower bond-angle residuals with little change in the *R* factors (Tronrud *et al.*, 2010 ▸; Tronrud & Karplus, 2011 ▸). The tests also revealed the rather striking result for ultrahigh-resolution structures that even those that had been refined using the conventional restraint library as target values had bond angles that agreed more closely with the CDL. As ultrahigh-resolution analyses allow the most accurate bond-angle determinations, this provided powerful validation for the greater accuracy of the CDL.

The incorporation of the CDL v.1.2 into *Phenix* (Adams *et al.*, 2010 ▸) allowed it to be tested in a re-refinement of the entire Protein Data Bank (Moriarty, Tronrud *et al.*, 2014 ▸). This not only confirmed that the CDL consistently provided much better bond-angle ideality, but also showed that on average there was even a slight improvement in the *R* factors: a slight lowering of *R*_free_ combined with a slight increase in *R*_work_ (see the inset in Fig. 2B in Moriarty, Tronrud *et al.*, 2014 ▸). This study also showed that the greater intrinsic accuracy of the CDL was already observable in structures determined at resolutions better than about 2 Å, at which point the backbone bond angles began to agree better with the CDL than with the conventional library against which they were restrained (see Fig. 2B of Moriarty, Tronrud *et al.*, 2014 ▸). For the N—C^α^—C bond angle, the crossover point occurred at an even more remarkable ∼3 Å resolution. In general, using the CDL v.1.2 decreased overall backbone bond-angle residuals by about 30% at all resolutions and decreased the N—C^α^—C bond-angle residuals by about 50% (Moriarty, Tronrud *et al.*, 2014 ▸).

Details of the implementation of the CDL into *Phenix* are presented in Moriarty, Tronrud *et al.* (2014 ▸), but here we find it useful to briefly note a few things. The first is that the restraints used are available for inspection in the Python dictionary object and incorporation into other applications following the simple example of the Python program *mmtbx.cdl_lookup* (Moriarty, Adams *et al.*, 2014 ▸) in the open-source *cctbx* (Grosse-Kunstleve *et al.*, 2002 ▸), which will display the restraints for a triplet of amino acids and pair of backbone angles. Secondly, because the restraints are conformation-dependent, the target values are updated every macrocycle based on the new coordinates with special consideration of the alternative locations. Thirdly, the weights are determined just as for any other library used in *Phenix*: the target standard deviations provide a unique weight for each restraint, and the optimal overall weight by which these are scaled is automatically determined by *Phenix* using a complex algorithm (Afonine *et al.*, 2011 ▸). Also, just as for other restraint libraries, users can override the automatically determined overall weight.

Despite the excellent performance of the backbone CDL v.1.2, before making it the default in *Phenix* we decided that it was important to verify that no significant problems would be caused by refining a protein against the CDL and then validating it against the conventional library that is currently used in most of the standard validation tools. In the transition period from validation with standard restraint libraries to the CDL library, it would be unfortunate if improved structures refined in *phenix.refine* (Afonine *et al.*, 2012 ▸) were flagged as being of poor stereochemical quality during deposition in the Protein Data Bank. Therefore, we took the ∼23 000 structures that we had previously refined with the CDL (Moriarty, Tronrud *et al.*, 2014 ▸) and validated each one using *MolProbity* (Chen *et al.*, 2010 ▸). Like all software based on *cctbx* (Grosse-Kunstleve *et al.*, 2002 ▸), *MolProbity* was easily loaded with CDL targets so that the CDL-based r.m.s.d. calculations of the bond and angle residuals could be directly compared with the validation results based on the Engh and Huber single-value library (SVL; *i.e.* conformation-independent) restraints that are the default in *MolProbity* and are also used by the PDB validation software. It should be noted that upcoming releases of the *MolProbity* web services and all programs in *Phenix* that use a model, including geometry idealization, will be able to make use of the CDL restraints for structures refined against the CDL.

As expected, in this head-to-head validation comparison, the overall bond-angle r.m.s.d. values are somewhat higher when validating a CDL-refined structure against the Engh and Huber restraints as opposed to the CDL restraints (Fig. 1 ▸ *a*). Encouragingly, the increase is only in the 0.3–0.4° range in a relatively resolution-independent manner. Furthermore, the CDL values are low enough that the higher r.m.s.d. values against the Engh and Huber restraints still have quite acceptable overall values of ∼1.7° or lower and so would not raise concerns in validation. Even more encouragingly, analysis of the backbone bond angles (*i.e.* the subset of angles that have differing targets in the two libraries) shows that the CDL-based residuals are even lower and the deviations from the Engh and Huber targets remain below 1.25° (solid lines in Fig. 1 ▸ *a*). We also assessed the difference in the bond-length r.m.s.d. values and found, as expected, virtually no difference (Fig. 1 ▸ *b*). For the bond angles, to determine whether outliers might cause a problem even if the overall deviations do not, we analysed the numbers of individual 6σ outliers. As shown in Figs. 1 ▸(*c*) and 1 ▸(*d*), there is very little change in 6σ bond-angle or bond-length outliers when validating a CDL-refined structure with SVL values compared with the CDL validation. Important to note is that because this is an unfiltered set of PDB entries, some of the structures have regions of the model that are poorly fitted and in fact should be outliers.

The improved geometry provided by the backbone CDL, in combination with the positive results from our validation analysis, led us to make the CDL v.1.2 the default in *Phenix* starting with release v.1.10-2155. We remind users that this library only defines conformation-dependent target backbone values for residues linked by *trans*-peptide bonds, so that residues linked by *cis*-peptide bonds and the side-chain bond lengths and angles are unchanged and are still based on the conventional restraint library of Engh & Huber (2001 ▸). For users that wish to use the Engh and Huber library instead of the CDL, the cdl=False option is available.

This change in the default library in *Phenix* moves the backbone CDL into the protein-modeling mainstream. This step represents a breach of a conceptual barrier, as it will stimulate people to move beyond the mindset of a ‘single ideal value’ paradigm to a more general ‘context-dependent’ ideal value paradigm in which the backbone conformation is just one example of a context that could influence geometry. This step also represents the breaching of a practical barrier by showing how an existing software framework can be adapted to accommodate the more complex ‘context-dependent’ paradigm. Obviously, updating validation tools to be able to use these more accurate target values is a key next step for continuing the transition, and we hope that the incorporation of the CDL into other crystallographic refinement and protein-modeling programs will follow, so that they can yield structures benefitting from this advance. The success of the CDL in improving model quality also should stimulate work to create further empirical conformation-dependent libraries that account for the rarer but still important residues with *cis*-peptide bonds and also that account for the variations in side-chain geometry that undoubtedly exist as a function of backbone and side-chain conformation. We have begun work to create a CDL for *cis*-peptides, and the fact that many fewer observations are available raises unique challenges that must be solved.

## Figures and Tables

**Figure 1:**
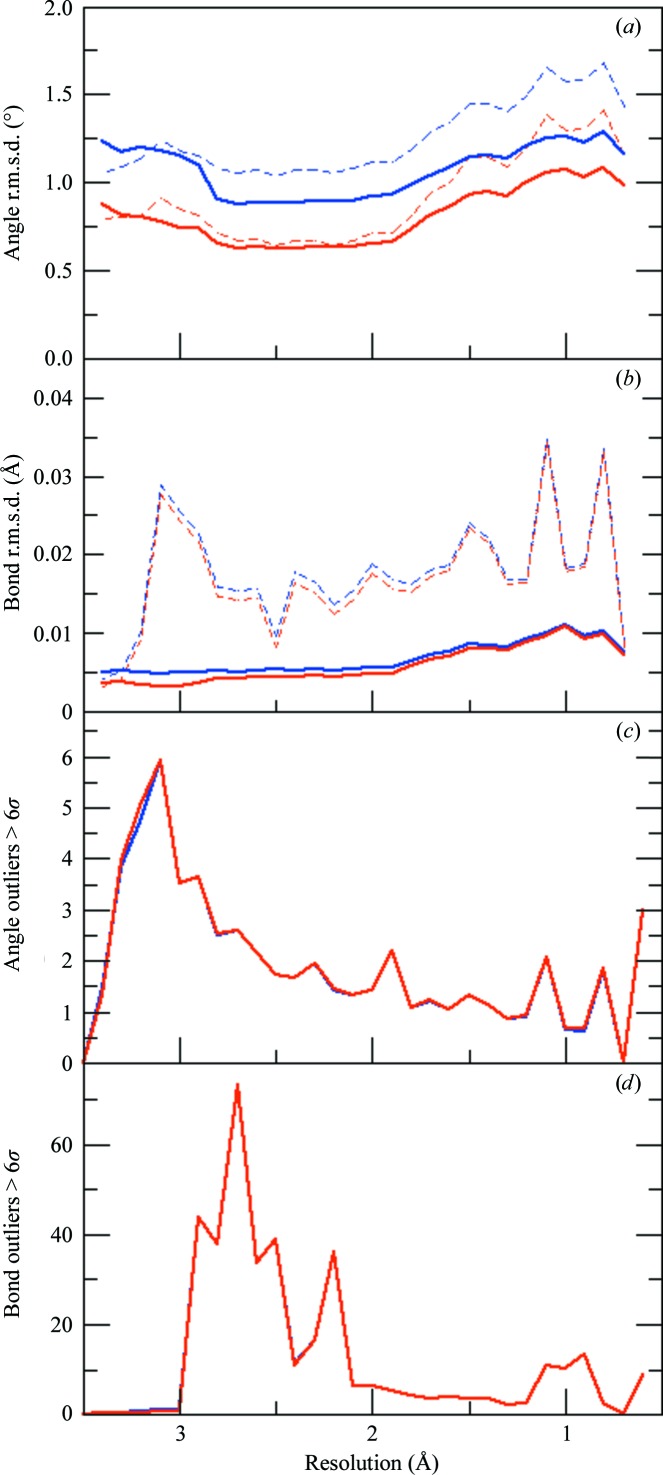
Comparison of the validation results for ∼23 000 re-refined PDB entries based on either the CDL or the Engh and Huber SVL restraints. Bins corresponding to 0.6, 0.7, 3.4, 3.5 and 3.6 Å have less than 50 observations. (*a*) Bond-angle r.m.s.d. values as a function of resolution for structures refined using the CDL v.1.2 and validated against the conventional SVL (blue lines) or against the CDL (red lines). The dashed lines are the values for the entire proteins, while the solid lines are based only on the backbone bond angles that are unique to the CDL. (*b*) Same as (*a*) but for bond lengths. (*c*) Average number of bond angles per structure as a function of resolution that are more than 6σ away from the restraint target value based on validation using either the conventional SVL (blue lines) or the CDL (red lines). (*d*) Same as (*c*) but for bond lengths. In (*c*) and (*d*) where the blue lines are not visible they are underneath the red lines.

## References

[bb1] Adams, P. D. *et al.* (2010). *Acta Cryst.* D**66**, 213–221.

[bb2] Afonine, P. V., Grosse-Kunstleve, R. W., Echols, N., Headd, J. J., Moriarty, N. W., Mustyakimov, M., Terwilliger, T. C., Urzhumtsev, A., Zwart, P. H. & Adams, P. D. (2012). *Acta Cryst.* D**68**, 352–367. 10.1107/S0907444912001308 PMC3322595 22505256

[bb3] Afonine, P. V., Echols, N., Grosse-Kunstleve, R. W., Moriarty, N. W. & Adams, P. D. (2011). *Comput. Crystallogr. Newsl.* **2**, 99–103.

[bb4] Berkholz, D. S., Shapovalov, M. V., Dunbrack, R. L. Jr & Karplus, P. A. (2009). *Structure*, **17**, 1316–1325. 10.1016/j.str.2009.08.012 PMC2810841 19836332

[bb5] Chen, V. B., Arendall, W. B., Headd, J. J., Keedy, D. A., Immormino, R. M., Kapral, G. J., Murray, L. W., Richardson, J. S. & Richardson, D. C. (2010). *Acta Cryst.* D**66**, 12–21. 10.1107/S0907444909042073 PMC2803126 20057044

[bb6] Dauter, Z. & Wlodawer, A. (2009). *Structure*, **17**, 1278–1279. 10.1016/j.str.2009.09.002 19836327

[bb7] Engh, R. A. & Huber, R. (2001). *International Tables for Crystallo­graphy*, Vol. *F*, edited by M. G. Rossmann & E. Arnold, pp. 382–392. Dordrecht: Kluwer Academic Publishers.

[bb8] Grosse-Kunstleve, R. W., Sauter, N. K., Moriarty, N. W. & Adams, P. D. (2002). *J. Appl. Cryst.* **35**, 126–136.

[bb9] Jiang, X., Cao, M., Teppen, B. J., Newton, S. Q. & Schäfer, L. (1995). *J. Phys. Chem*, **99**, 10521–10525.

[bb10] Karplus, P. A. (1996). *Protein Sci.* **5**, 1406–1420. 10.1002/pro.5560050719 PMC2143451 8819173

[bb11] Karplus, P. A., Shapovalov, M. V., Dunbrack, R. L. & Berkholz, D. S. (2008). *Acta Cryst.* D**64**, 335–336. 10.1107/S0907444908002333 18323629

[bb12] Moriarty, N. W., Adams, P. D. & Karplus, P. A. (2014). *Comput. Crystallogr. Newsl.* **5**, 42–49.

[bb13] Moriarty, N. W., Tronrud, D. E., Adams, P. D. & Karplus, P. A. (2014). *FEBS J.* **281**, 4061–4071. 10.1111/febs.12860 PMC4169323 24890778

[bb14] Scarsdale, J. N., Van Alsenoy, C., Klimkowski, V. J., Schaefer, L. & Momany, F. A. (1983). *J. Am. Chem. Soc.* **105**, 3438–3445.

[bb15] Schäfer, L., Klimkowski, V. J., Momany, F. A., Chuman, H. & Van Alsenoy, C. (1984). *Biopolymers*, **23**, 2335–2347.

[bb16] Sheldrick, G. M. (2008). *Acta Cryst.* A**64**, 112–122. 10.1107/S0108767307043930 18156677

[bb17] Tronrud, D. E., Berkholz, D. S. & Karplus, P. A. (2010). *Acta Cryst.* D**66**, 834–842. 10.1107/S0907444910019207 PMC2897700 20606264

[bb18] Tronrud, D. E. & Karplus, P. A. (2011). *Acta Cryst.* D**67**, 699–706. 10.1107/S090744491102292X PMC3144852 21795811

[bb19] Tronrud, D. E., Ten Eyck, L. F. & Matthews, B. W. (1987). *Acta Cryst.* A**43**, 489–501.

